# Adverse and Serious Adverse Events Associated With Tocilizumab in COVID-19 Pneumonia and With Cyclosporin, Pazopanib, and Insulin Glargine in Other Diseases: A Single-Center Cross-Sectional Study From a Tertiary Care Center in Bangladesh

**DOI:** 10.7759/cureus.105738

**Published:** 2026-03-23

**Authors:** Kawser Ahmed, Arsalan Bangash, Yaseen Hussain, Faaraan Bangash, Ahmed Reza Suny, A B M Abdullah

**Affiliations:** 1 Acute Internal Medicine, Ipswich Hospital, Ipswich, GBR; 2 Internal Medicine, Dhaka Medical College Hospital, Dhaka, BGD; 3 General Medicine, Northampton General Hospital, Northampton, GBR; 4 Acute Medicine, Northampton General Hospital, Northampton, GBR; 5 Surgery, Bangladesh Institute of Research and Rehabilitation in Diabetes, Endocrine and Metabolic Disorders (BIRDEM) General Hospital, Dhaka, BGD; 6 Surgery, Bangladesh-Korea Friendship Hospital, Savar, BGD

**Keywords:** covid-19, cyclosporine, insulin glargine, pazopanib, tocilizumab

## Abstract

Objective

This study aimed to evaluate reported adverse events (AEs) and serious adverse events (SAEs) associated with tocilizumab in COVID-19 pneumonia and with cyclosporine, insulin glargine, and pazopanib in other clinical indications using real-world pharmacovigilance data.

Methods

This cross-sectional observational study analyzed suspected SAE reports submitted to the National Pharmacovigilance Centre (NPC), Directorate General of Drug Administration (DGDA), Bangladesh, between March and July 2020 from a tertiary care center in Bangladesh. A total of 60 patients were included. Of these, 46 received tocilizumab for COVID-19 pneumonia, and 14 received cyclosporine (n=5), insulin glargine (n=4), or pazopanib (n=5) for other indications. Incomplete reports were excluded to minimize bias. Descriptive statistics and association analyses were performed to evaluate patient characteristics, outcomes, and dose-outcome relationships.

Results

After the exclusion of incomplete records, 28 patients treated with tocilizumab were analyzed. The majority were male (25/28, 89.29%), with a mean age of 59.75±15.23 years. SAEs occurred in 27 patients (96.43%), predominantly categorized as "other medically important conditions", and one death (1/28, 3.57%) was reported. All five patients receiving cyclosporine experienced fatal outcomes (5/5, 100%). Fatal outcomes were observed in 3/4 patients (75%) receiving insulin glargine and in 2/5 patients (40%) receiving pazopanib. The results reflect outcomes among reported SAEs; however, they should not be interpreted as statistically significant.

Conclusion

This study highlights serious adverse outcomes associated with tocilizumab and other commonly used medications in real-world clinical practice. Although casualty cannot be established due to the limited sample size and observational design, the findings emphasize the importance of vigilant pharmacovigilance and cautious interpretation of treatment outcomes.

## Introduction

COVID-19, caused by severe acute respiratory syndrome coronavirus-2 (SARS-CoV-2), has been associated with a wide spectrum of clinical severity, ranging from mild respiratory symptoms to severe pneumonia, acute respiratory distress syndrome, multiorgan failure, and death [1]. A dysregulated host immune response, particularly the cytokine release syndrome mediated by interleukin-6 (IL-6), plays a central role in severe COVID-19 pneumonia. Tocilizumab, a monoclonal antibody targeting the IL-6 receptor, has therefore been repurposed as an anti-inflammatory therapy in hospitalized severe COVID-19 patients. Although several clinical trials and observational studies have evaluated its efficacy, real-world safety data, especially from low- and middle-income countries, remain limited, and concerns persist regarding adverse events (AEs) and serious adverse events (SAEs) [2].

Beyond COVID-19, pharmacovigilance of widely used agents such as cyclosporine, pazopanib, and insulin glargine is equally important. Cyclosporine, a calcineurin inhibitor, is commonly used as an immunosuppressant in transplant recipients and hematologic malignancies; insulin glargine is a long-acting insulin analog used in diabetes mellitus; pazopanib, a tyrosine kinase inhibitor, is prescribed for renal cell carcinoma and other malignancies. Each of these agents carries a distinct safety profile, yet data on serious adverse outcomes from routine clinical practice are often underreported [3-6]. 

This cross-sectional study aims to describe and analyze reported AEs and SAEs associated with tocilizumab in COVID-19 pneumonia, as well as cyclosporine, pazopanib, and insulin glargine in different disease conditions, using spontaneous adverse drug reaction reports submitted to the National Pharmacovigilance Centre (NPC), Directorate General of Drug Administration (DGDA), Bangladesh. By highlighting real-world safety signals, this study seeks to contribute to improved understanding and monitoring of these commonly used medications.

## Materials and methods

Study design and data source

This was a cross-sectional observational study based on suspected SAE reports submitted to the NPC, DGDA, Bangladesh, between March and July 2020.

All reports included in this analysis originated from Dhaka Medical College Hospital, Dhaka, Bangladesh, which functions as a tertiary care referral center and forwards adverse drug reaction reports to the national pharmacovigilance study rather than a national database analysis. 

Study population

**Figure 1:**
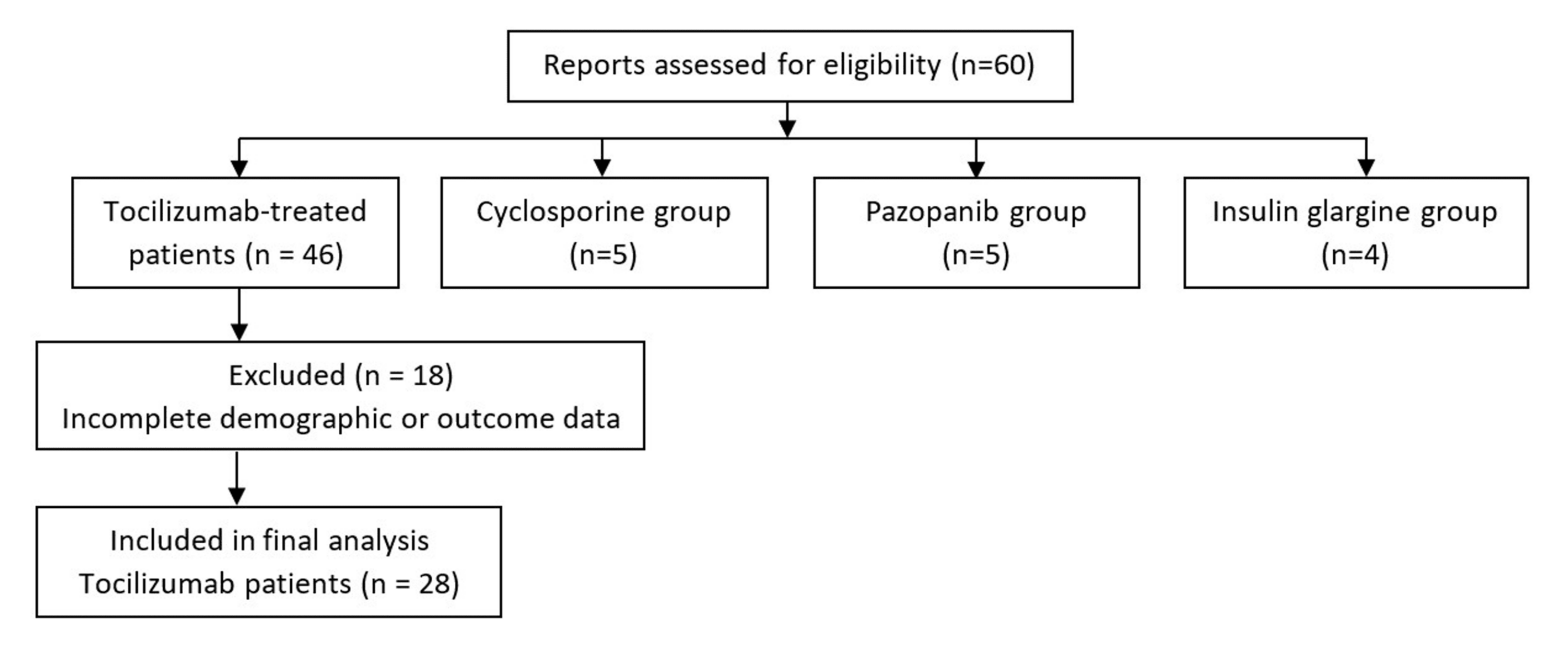
Cohort flow diagram of patient selection This figure illustrates the selection of suspected serious adverse event reports assessed for eligibility, the exclusion of incomplete records, and the final distribution of patients across treatment groups: tocilizumab (n=28), cyclosporine (n=5), pazopanib (n=5), and insulin glargine (n=4).

A total of 60 patients were initially identified. Forty-six patients received tocilizumab for COVID-19 pneumonia, while 14 patients received cyclosporine, insulin glargine, or pazopanib for other indications. Reports with incomplete demographic or outcome data were excluded to reduce reporting bias (Figure 1).

Inclusion and exclusion criteria

Patients were included if they had a suspected SAE report submitted to the NPC, DGDA, Bangladesh, between March and July 2020 and had received tocilizumab, cyclosporine, insulin glargine, or pazopanib for any clinical indication. Only reports with available information on patient demographics, drug exposure, seriousness of AEs, and clinical outcomes were considered eligible for analysis. 

Reports were excluded if key demographic data, drug details, seriousness classification, or outcome information were missing or incomplete. Duplicate reports and follow-up reports referring to the same patient were also excluded from the final analysis. 

Variables and definitions

Data extracted included age, sex, drug name, daily dose, duration of therapy, indication, seriousness of AEs, and outcomes (recovered, not recovered, unknown, or fatal). Seriousness was defined according to standard pharmacovigilance criteria, including death, hospitalization, and other medically important conditions.

Statistical analysis

Descriptive statistics were used to summarize patients' characteristics. Associations between demographic or dosing variables and seriousness or outcomes were evaluated. A p-value of <0.05 was considered statistically significant.

## Results

Tocilizumab

Data were collected from a summary sheet of suspected SAE reports submitted to the NPC, DGDA, Bangladesh, between March and July 2020 from a single tertiary care center in Dhaka, Bangladesh. Initially, data from 46 patients with COVID-19 pneumonia were identified. However, 18 reports were incomplete and were excluded to minimize reporting bias. Therefore, 28 patients were included in the final analysis. 

Among the 28 evaluable patients treated with tocilizumab, 25 (89.29%) were male, and three (10.71%) were female. The minimum age was 27 years, while the maximum age was 85 years, with a mean age of 59.75±15.23 years. All patients received a daily dose of 8 mg/kg body weight. 

SAEs classified as "other medically important conditions" occurred in 27 patients (96.43%). These events primarily included secondary bacterial or fungal infection, sepsis, significant elevation of liver transaminases, acute kidney injury, and infusion-related hypersensitivity reactions. The most frequently reported clinical features were hospital-acquired pneumonia, septic shock, and biochemical hepatitis. One fatal outcome (1/28, 3.57%) was reported. No inferential statistical testing of dose-outcome associations was performed, as all patients received a uniform dose and the dataset was analyzed descriptively. Of note, the NPC summary sheets did not consistently provide granular clinical descriptors of these events. Therefore, further subclassification of these "medically important conditions" was not possible in this dataset, limiting the clinical interpretability of this category (Table 1).

**Table 1:** Summary of suspected SAEs reported with tocilizumab (Actemra) to NPC, DGDA, Bangladesh, from March to July 2020 This table summarizes suspected SAEs associated with tocilizumab (Actemra) reported to the NPC, DGDA, Bangladesh, from March to July 2020. Data include patient demographics (age, sex), drug details (brand name, generic name, daily dose, and duration), indication, seriousness of the event, and outcome/status. SAEs: serious adverse events; NPC: National Pharmacovigilance Centre; DGDA: Directorate General of Drug Administration

Sl. no.	Age	Sex	Brand name	Generic name	Daily dose (mg)	Duration (month)	Indication	Seriousness	Outcome/status
1	44	Male	Actemra	Tocilizumab	8 mg/kg	2 days	COVID-19 pneumonia	Other medically important	Unknown
2	67	Female	Actemra	Tocilizumab	8 mg/kg	1 day	COVID-19 pneumonia	Other medically important	Unknown
3	50	Male	Actemra	Tocilizumab	8 mg/kg	1 day	COVID-19 pneumonia	Other medically important	Unknown
4	67	Male	Actemra	Tocilizumab	8 mg/kg	1 day	COVID-19 pneumonia	Death	Fatal
5	58	Male	Actemra	Tocilizumab	8 mg/kg	2 days	COVID-19 pneumonia	Other medically important	Unknown
6	68	Male	Actemra	Tocilizumab	8 mg/kg	2 days	COVID-19 pneumonia	Other medically important	Unknown
7	45	Male	Actemra	Tocilizumab	8 mg/kg	1 day	COVID-19 pneumonia	Other medically important	Unknown
8	40	Male	Actemra	Tocilizumab	8 mg/kg	2 days	COVID-19 pneumonia	Other medically important	Unknown
9	70	Male	Actemra	Tocilizumab	8 mg/kg	3 days	COVID-19 pneumonia	Other medically important	Unknown
10	84	Female	Actemra	Tocilizumab	8 mg/kg	4 days	COVID-19 pneumonia	Other medically important	Unknown
11	60	Male	Actemra	Tocilizumab	8 mg/kg	5 days	COVID-19 pneumonia	Other medically important	Unknown
12	56	Female	Actemra	Tocilizumab	8 mg/kg	4 days	COVID-19 pneumonia	Other medically important	Unknown
13	85	Male	Actemra	Tocilizumab	8 mg/kg	June 18 to ongoing	COVID-19 pneumonia	Other medically important	Unknown
14	75	Male	Actemra	Tocilizumab	8 mg/kg	June 16 to ongoing	COVID-19 pneumonia	Other medically important	Unknown
15	80	Male	Actemra	Tocilizumab	8 mg/kg	June 17 to ongoing	COVID-19 pneumonia	Other medically important	Unknown
16	27	Male	Actemra	Tocilizumab	8 mg/kg	June 16 to ongoing	COVID-19 pneumonia	Other medically important	Unknown
17	71	Male	Actemra	Tocilizumab	8 mg/kg	June 17 to ongoing	COVID-19 pneumonia	Other medically important	Unknown
18	47	Male	Actemra	Tocilizumab	8 mg/kg	June 17 to ongoing	COVID-19 pneumonia	Other medically important	Unknown
19	68	Male	Actemra	Tocilizumab	8 mg/kg	June 16 to ongoing	COVID-19 pneumonia	Other medically important	Unknown
20	45	Male	Actemra	Tocilizumab	8 mg/kg	3 days	COVID-19 pneumonia	Other medically important	Unknown
21	75	Male	Actemra	Tocilizumab	8 mg/kg	June 22 to ongoing	COVID-19 pneumonia	Other medically important	Unknown
22	31	Male	Actemra	Tocilizumab	8 mg/kg	June 22 to ongoing	COVID-19 pneumonia	Other medically important	Unknown
23	53	Male	Actemra	Tocilizumab	8 mg/kg	June 22 to ongoing	COVID-19 pneumonia	Other medically important	Unknown
24	52	Male	Actemra	Tocilizumab	8 mg/kg	June 21 to ongoing	COVID-19 pneumonia	Other medically important	Unknown
25	49	Male	Actemra	Tocilizumab	8 mg/kg	June 21 to ongoing	COVID-19 pneumonia	Other medically important	Unknown
26	70	Male	Actemra	Tocilizumab	8 mg/kg	June 20 to ongoing	COVID-19 pneumonia	Other medically important	Unknown
27	65	Male	Actemra	Tocilizumab	8 mg/kg	2 days	COVID-19 pneumonia	Other medically important	Unknown
28	71	Male	Actemra	Tocilizumab	8 mg/kg	2 days	COVID-19 pneumonia	Other medically important	Unknown

Cyclosporine

Five patients received cyclosporine for acute leukemia or kidney transplant-related indications. The mean age was 44.2±25.39 years. Four patients (4/5, 80%) were male, and one patient (1/5, 20%) was female. Fatal outcome occurred in all five patients (5/5, 100%). Given the very small sample size (n=5), no inferential statistical testing was performed for the cyclosporine subgroup. Findings were therefore presented descriptively as a case series. No associations were identified among age, sex, or daily dose and clinical outcomes (Table 2).

**Table 2:** Summary of suspected SAEs reported with cyclosporine (Sandimmun Neoral) This table summarizes suspected SAEs associated with cyclosporine (Sandimmun Neoral) use. Patient demographics (age, sex), drug details (brand name, generic name, daily dosing), indication for use, seriousness of the event, and outcome/status are presented. All reported cases in this dataset resulted in fatal outcomes. SAEs: serious adverse events

Sl. no.	Age	Sex	Brand name	Generic name	Daily dosing	Indication	Seriousness	Outcome/status
1	25	Male	Sandimmun Neoral	Cyclosporine	100 mg	Acute leukemia	Death	Fatal
2	72	Male	Sandimmun Neoral	Cyclosporine	150 mg	Prophylaxis, kidney transplant	Death	Fatal
3	25	Male	Sandimmun Neoral	Cyclosporine	100 mg	Acute leukemia	Death	Fatal
4	72	Male	Sandimmun Neoral	Cyclosporine	150 mg	Prophylaxis, kidney transplant	Death	Fatal
5	27	Female	Sandimmun Neoral	Cyclosporine	25 mg	Post-right kidney transplantation	Death	Fatal

Pazopanib

Five male patients received pazopanib for renal carcinoma, soft tissue sarcoma, or spinal cord tumors. The mean age was 50.2±22.55 years. All patients received a daily dose of 200 mg. Fatal outcomes occurred in two patients (2/5, 40%), while three patients (3/5, 60%) recovered. Due to the small sample size (n=5), outcomes in patients receiving pazopanib are presented descriptively as a case series. No inferential statistical analyses were performed (Table 3). 

**Table 3:** Summary of suspected SAEs reported with pazopanib (Votrient) This table summarizes suspected SAEs reported with pazopanib (Votrient). Data include patient demographics (age, sex), drug details (brand name, generic name, daily dose), indication for use, seriousness of the event, and outcome/status. Outcomes ranged from recovery to death. SAEs: serious adverse events

Sl. no.	Age	Sex	Brand name	Generic name	Daily dose (mg)	Indication	Seriousness	Outcome/status
1	65	Male	Votrient	Pazopanib	200	Renal cancer	Hospitalization	Recovered
2	65	Male	Votrient	Pazopanib	200	Renal cancer	Hospitalization	Recovered
3	65	Male	Votrient	Pazopanib	200	Renal cancer	Hospitalization	Recovered
4	42	Male	Votrient	Pazopanib	200	Soft tissue sarcoma	Death	Fatal
5	14	Male	Votrient	Pazopanib	200	Tumor in the spinal cord	Death	Fatal

Insulin glargine

Four patients received insulin glargine for diabetes mellitus or type 2 diabetes mellitus. The mean age was 57.5±8.81 years, with equal sex distribution. Among the four reports of SAEs involving insulin glargine, three resulted in fatal outcomes (3/4, 75%), while one patient (1/4, 25%) recovered. Given the very small sample size (n=4), no inferential statistical testing was performed for the insulin glargine subgroup. Findings are therefore presented descriptively as a case series (Table 4). 

**Table 4:** Summary of suspected SAEs reported with insulin (insulin glargine) This table summarizes suspected SAEs reported with insulin glargine. Data include patient demographics (age, sex), drug details (brand name, generic name, daily dose), indication, seriousness of the event, and outcome/status. Outcomes ranged from recovery to death. SAEs: serious adverse events

Sl. no.	Age	Sex	Brand name	Generic name	Daily dose (mg/U)	Indication	Seriousness	Outcome/status
1	53	Female	Lantus-Solo Star pen	Insulin glargine	20 U	Diabetes mellitus	Other medically important	Recovered
2	50	Male	Lantus by All Star pen	Insulin glargine	16 U	Diabetes mellitus	Other medically important	Fatal
3	70	Female	Lantus	Insulin glargine	Injection	Type 2 diabetes mellitus	Death	Fatal
4	57	Male	Lantus	Insulin glargine	10 U	Type 2 diabetes mellitus	Death	Fatal

## Discussion

This cross-sectional pharmacovigilance study describes suspected AEs and SAEs associated with tocilizumab use in COVID-19 pneumonia and with cyclosporine, pazopanib, and insulin glargine in other clinical indications, using spontaneous reports submitted to the NPC, DGDA, Bangladesh, between March and July 2020. Importantly, all reports analyzed in this study originated from a single tertiary care center (Dhaka Medical College Hospital, Dhaka); therefore, the findings should not be interpreted as nationally representative. Nevertheless, the study provides real-world safety signals from a low- and middle-income country during the early phase of the COVID-19 pandemic, a period characterized by rapid therapeutic repurposing and limited clinical evidence.

Tocilizumab and COVID-19 pneumonia

Tocilizumab, an interleukin-6 receptor antagonist, was widely investigated during the COVID-19 pandemic to mitigate cytokine-mediated hyperinflammation. Large randomized controlled trials, including RECOVERY AND REMAP-CAP, demonstrated mortality reduction and decreased progression to mechanical ventilation in selected patients with systemic inflammation. However, these trials primarily evaluated efficacy and did not focus extensively on AE patterns in real-world clinical practice [7-10].

In the present study, 27 patients (96.43%) receiving tocilizumab experienced events classified as "other medically important conditions", with a reported fatality rate of 3.57%. These figures should be interpreted cautiously, as the dataset was derived exclusively from suspected SAE reports, inherently enriching the sample with severe outcomes. Therefore, the observed frequency of SAEs does not represent the overall safety profile of tocilizumab among all treated patients [10].

No inferential statistical testing of dose-outcome associations was performed, as all patients received a uniform weight-based dose (8 mg/kg) and the dataset was analyzed descriptively. Although secondary infections, hepatic dysfunction, or hematologic abnormalities were frequently observed, these complications are well-recognized in critically ill patients receiving immunomodulatory therapy and cannot be causally attributed to tocilizumab in this cross-sectional pharmacovigilance analysis. Though one fatal outcome occurred in a patient receiving the recommended dose, the study design and lack of systematic data on disease severity, comorbidities, and concomitant therapies preclude causal inference. 

Compared with existing literature, these findings reinforce the need for cautious patient selection and close monitoring when administering tocilizumab, especially in resource-limited settings where advanced supportive care and laboratory monitoring may be constrained.

Cyclosporine-related outcomes

Cyclosporine is a calcineurin inhibitor widely used in transplant medicine and hematologic malignancies. In this study, all five patients receiving cyclosporine experienced fatal outcomes. While this observation appears striking, it must be contextualized within the clinical characteristics of the population. Patients receiving cyclosporine in this dataset had severe underlying conditions, including acute leukemia and post-kidney transplantation, which are associated with high baseline mortality independent of pharmacotherapy [11].

Previous studies have not reported universal fatality associated with cyclosporine use. Instead, known risks include nephrotoxicity, infection, and metabolic complications. The lack of significant association between age, sex, or daily dose and seriousness in this study suggests that disease severity and immunocompromised status were likely the primary drivers of adverse outcomes rather than cyclosporine exposure alone. Nonetheless, the findings highlight the importance of vigilant pharmacovigilance in high-risk patient populations [3].

Pazopanib and cancer patients

Pazopanib, a multitargeted tyrosine kinase inhibitor, is associated with known adverse effects, including hepatotoxicity, hypertension, and cardiovascular events. In this study, a fatality rate of 40% was observed among patients receiving pazopanib, with deaths occurring in individuals with soft tissue sarcoma and spinal cord tumors [12]. Clinical trials of pazopanib generally report lower rates of fatal AEs, suggesting that the outcomes observed here may reflect advanced disease stage, limited treatment options, or inadequate supportive care rather than unexpected drug toxicity. Nonetheless, the presence of both recovered and fatal outcomes at a uniform dosing regimen highlights interpatient variability and the importance of individualized risk assessment [13]. 

Insulin glargine safety signals

Insulin glargine is a long-acting insulin analog with a well-established safety profile. In this study, fatal outcomes were reported in 75% of insulin glargine cases, a finding that contrasts sharply with large-scale epidemiological data. This discrepancy is likely attributable to reporting bias, as SAE reports involving insulin often occur in the context of severe hypoglycemia, advanced diabetic complications, or significant comorbid illness. A slight association between indication and seriousness was observed (p=0.046), further supporting the influence of underlying disease rather than drug toxicity. These results underscore a key principle in pharmacovigilance research: spontaneous reporting systems are designed to detect safety signals, not to estimate incidence or establish causality [14].

Significance of the study

This study contributes to the existing literature by providing pharmacovigilance data from Dhaka Medical College Hospital, Dhaka, Bangladesh, a region underrepresented in global drug safety research. During the early COVID-19 pandemic, rapid drug repurposing occurred in the absence of robust safety data, particularly in low- and middle-income country settings. By analyzing spontaneous AE reports, this study captures real-world safety concerns that may not be fully reflected in randomized trials.

Furthermore, the study highlights systemic challenges in AE reporting, including incomplete data, unknown outcomes, and limited clinical details. Addressing these gaps is essential for strengthening national pharmacovigilance systems and improving patient safety.

Limitations

Several limitations should be acknowledged. The cross-sectional design precludes causal inference. The sample size was small, particularly for cyclosporine, insulin glargine, and pazopanib. The reliance on spontaneous reports introduces reporting bias and underreporting. Additionally, critical clinical variables such as disease severity, laboratory parameters, and concomitant medications were unavailable.

Learning points

This study demonstrates that a high seriousness rate in spontaneous AE reports reflects the nature of the reporting mechanism rather than the true incidence of adverse outcomes. Dose-related safety signals, such as those observed with tocilizumab, warrant cautious interpretation and further investigation in larger, well-designed studies. Underlying disease severity remains a major confounder in AE reporting and must be carefully considered when interpreting safety data. Pharmacovigilance studies are primarily hypothesis-generating and complement, but do not replace, evidence from randomized controlled trials. Strengthening the AE reporting system is essential, particularly in low- and middle-income countries, to improve drug safety surveillance and patient outcomes. 

## Conclusions

This cross-sectional pharmacovigilance study provides valuable real-world insights into suspected AEs associated with tocilizumab, cyclosporine, pazopanib, and insulin glargine. While causality cannot be established, the findings underscore the importance of ongoing drug safety monitoring and cautious interpretation of AE data, particularly during public health emergencies and in resource-limiting setting.
